# Molecular characterization of *Klebsiella pneumoniae* isolates from stool specimens of outpatients in sentinel hospitals Beijing, China, 2010–2015

**DOI:** 10.1186/s13099-017-0188-7

**Published:** 2017-06-30

**Authors:** Bing Lu, Haijian Zhou, Xin Zhang, Mei Qu, Ying Huang, Quanyi Wang

**Affiliations:** 1 Institute for Infectious Disease and Endemic Disease Control, Beijing Center for Disease Prevention and Control, Beijing Center for Prevention Medical Research, Beijing Key Laboratory of Diagnostic and Traceability Technologies for Food Poisoning, Beijing, 100013 China; 2 State Key Laboratory for Infection Disease Prevention and Control, National Institute for Communicable Disease Prevention and Control, Chinese Center for Disease Control and Prevention, Beijing, 102206 China; 3 Collaborative Innovation Center for Diagnosis and Treatment of Infectious Diseases, Hangzhou, China

**Keywords:** *Klebsiella pneumoniae*, Drug resistance, Multilocus sequence typing

## Abstract

**Background:**

*Klebsiella pneumoniae* (*K. pneumoniae*) is an opportunistic pathogen associated with community-acquired infections and nosocomial infections. From 2010 to 2015, *K. pneumoniae* testing was included into the exiting diarrhea-syndrome surveillance with objective to estimate the prevalence of *K. pneumoniae* in diarrhea-syndrome patients, test antibiotics susceptibility and investigate molecular characteristics.

**Methods:**

Stool specimens from diarrhea-syndrome outpatients were cultured and identified the pathogens by the Vitek2 Compact instrument. The isolated *K. pneumoniae* strains were tested for antibiotics susceptibility by minimal inhibitory concentration (MIC) method, and subtyped by pulsed field gel electrophoresis (PFGE) and multilocus sequence typing (MLST).

**Results:**

22 *K. pneumoniae* strains were identified from 4340 stool specimens of outpatients who visited sentinel hospitals in Beijing during 2010–2015. All strains were sensitive to gentamicin, nalidixic acid, ciprofloxacin, ceftriaxone, cefotaxime, cefepime, imipenem. The highest resistance rate of *K. pneumoniae* strains was 100% to amoxicillin–clavulanate, followed by 72.7% to ampicillin. These 22 *K. pneumoniae* strains were characterized into 21 different PFGE types and 20 MLST types with less similarity.

**Conclusions:**

The detection rate of *K. pneumoniae* in stool specimens from outpatients with diarrhea syndromes was about 0.5% in Beijing. Less similarity of the isolated strains indicated the unlikely long-term circulating of *K. pneumoniae* in the community. ST23 was the most common genotype. Drug resistance of the community-acquired *K. pneumoniae* was not a serious problem in comparing with hospital-acquired infections. High vigilance in the community-acquired *K. pneumoniae* strains and investigation of pathogens’ microbiological characteristics are valuable for signals detection for drug resistance in population and strains variation.

## Background

*Klebsiella* spp. is ubiquitous in nature. *Klebsiella pneumoniae (K. pneumoniae)* is an opportunistic pathogen associated with both community-acquired and nosocomial infections, causing pneumoniae, abscess, bacteremia, and urinary tract infections [1], and occasionally causes diarrhea in humans [2, 3].

The surveillance on diarrhea-syndrome outpatients included any age people in Beijing, involving 245 sentinel hospitals from all 16 districts. Stool specimens were collected from diarrhea-syndrome outpatients in sentinel hospitals [4], and tested the common diarrhea induced pathogens including rotavirus, norovirus, diarrheagenic *Escherichia coli*, *Salmonella,* and *Shigella* spp. [5]. Because the increasingly nosocomial infections caused by *K. pneumoniae* might impose an increasing risk of infections in communities, from 2010 to 2015, *K. pneumoniae* testing was included into the exiting diarrhea-syndrome surveillance with objective to estimate the prevalence of *K. pneumoniae* in diarrhea-syndrome outpatients, test antibiotics susceptibility and investigate microbiological characteristics.

## Methods

### Identification of bacterial strains

Stool specimens from diarrhea-syndrome outpatients were collected through Beijing diarrhea-syndrome surveillance from 2010 to 2015. Diarrheagenic bacteria strains were cultured and isolated from stool specimens firstly. Any isolated positive bacteria strains were further identified the pathogens (e.g., *Salmonella, Shigella* spp., diarrheagenic *Escherichia coli*, *Vibrio parahemolyticus*, and *K. pneumoniae)* by the Vitek2 Compact instrument (bioMérieux. Marcy, France). The isolated *K. pneumoniae* strains were proceeding for antibiotics susceptibility test and molecular characterization.

### Antibiotics susceptibility testing

Antibiotics susceptibility testing for the *K. pneumoniae* strains was measured by minimal inhibitory concentration (MIC) method. The MICs were interpreted by the standards of Clinical and Laboratory Standards Institute (CLSI) document M100-S26:2016 [6]. The following 14 antibiotics sourced from Shanghai Xingbai Co. (AST panel for aerobic gram negative bacilli) were used for antimicrobial susceptibility test: ciprofloxacin, ampicillin, amoxicillin–clavulanate, chloramphenicol, sulfisoxazole, trimethoprim–sulfamethoxazole, nalidixic acid, ceftriaxone, gentamicin, tetracycline, cefotaxime, cefoxitin, cefepime, imipenem. *Escherichia coli* strain ATCC 25922 was in use as quality-control strains for the antibiotics susceptibility testing. MIC level at or above 2 μg/mL for cefotaxime and ceftriaxone indicates the strains possibly produced extended-spectrum beta-lactamases (ESBL), which needs further confirmation. MIC for ceftazidime combination with clavulanate decrease at least three twofold concentration in contrast with the MIC value for ceftazidime alone (e.g., ceftazidime MIC = 8 μg/mL; ceftazidime–clavulanate MIC = 1 μg/mL) confirms the existing ESBL production strains [6].

### Pulse field gel electrophoresis

*K. pneumoniae* strains were subtyped by pulse field gel electrophoresis (PFGE) method using restriction endonucleases *Xba*I [7]. The restriction endonuclease *Xba*I (Fermentas) was used to digest the prepared genomic DNA and resultant DNA fragments were separated in a PFGE CHEF-DR III system (Bio-Rad Laboratories) in 0.5× Tris–borate–EDTA buffer at 120 V for 18.5 h, with pulse times ranging from 6 to 36 s. PFGE dendrogram was generated by BioNumerics version 7.1 (Applied Math, Belgium) with the unweighted pair-group method (UPGMA) and Dice coefficient. Isolates that exhibited a PFGE profile with more than 80% similarity were considered as closely related strains [8, 9].

### Multilocus sequence typing

Multilocus sequence typing (MLST) was performed to subtype *K. pneumoniae* strains using seven housekeeping genes (*gapA*, *infB*, *mdh*, *pgi*, *phoE*, *rpoB*, and *tonB*) [10]. Seven gene fragments of the *K. pneumoniae* strains were amplified by PCR and sequenced. The allele sequences and sequence types (STs) were determined by the Institute Pasteur Klebsiella MLST database (http://bigsdb.web.pasteur.fr/klebsiella/klebsiella.html). BioNumerics version 7.1 software was used to create the minimum spanning tree. In the minimum spanning tree, the founder ST was defined as the greatest number of single-locus variants. Types were represented by circles and the size of a circle indicated the number of strains with this particular type.

## Results

### Identification of bacterial strains

In the 4340 stool specimens collected in the surveillance period from 2010 to 2015, 22 *K. pneumoniae* strains were identified: one strain in 2010, three strains in 2011 and 2012 respectively, five strains in 2013, nine strains in 2014, and one strain in 2015; 16 out of 22 positive *K. pneumoniae* were isolated from April to October, while six positives identified in low-incidence seasons 2010–2015.

### Antibiotics susceptibility results

Antibiotics susceptibility is shown in Table 1. The result showed that 22 *K. pneumoniae* strains were all sensitive to gentamicin, nalidixic acid, ciprofloxacin, ceftriaxone, cefotaxime, cefepime, imipenem, followed by 95.5% to trimethoprim–sulfamethoxazole, cefoxitin, and 63.6% to sulfisoxazole. The highest resistance rate of *K. pneumoniae* strains was 100% to amoxicillin–clavulanate, and followed by 72.7% to ampicillin. Moreover, all 22 *K. pneumoniae* strains were sensitive to ceftriaxone, cefotaxime, the MIC was less than 1 μg/mL, and no ESBL production strain was identified.

**Table 1:** Antibiotics susceptibility results of 22 *K. pneumoniae* strains

Antibiotics agent	R (%)	I (%)	S (%)
Ampicillin	16 (72.7%)	6 (27.3%)	0
Amoxicillin–clavulanate	22 (100%)	0	0
Ceftriaxone	0	0	22 (100%)
Cefotaxime	0	0	22 (100%)
Cefepime	0	0	22 (100%)
Cefoxitin	1 (4.5%)	0	21 (95.5%)
Imipenem	0	0	22 (100%)
Tetracycline	2 (9.1%)	7 (31.8%)	13 (59.1%)
Nalidixic acid	0	0	22 (100%)
Ciprofloxacin	0	0	22 (100%)
Trimethoprim–sulfamethoxazole	1 (4.5%)	0	21 (95.5%)
Sulfisoxazole	8 (39.3%)	0	14 (63.6%)
Chloramphenicol	4 (18.2%)	7 (31.8%)	11 (50%)
Gentamicin	0	0	22 (100%)

*R* resistant, *I* intermediate, *S* susceptible

### PFGE typing

**Fig. 1:**
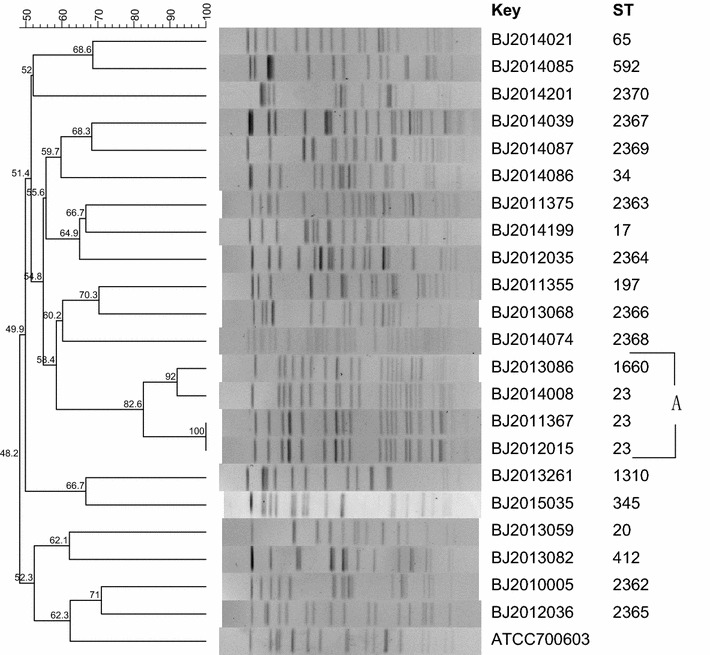
Dendrogram of PFGE pattern for 22 *K. pneumoniae* strains

22 isolates showed 21 different PFGE types. The dendrogram of the PFGE image showed that four *K. pneumoniae* strains isolated during 2011–2014 had over 80% similarity, which categorized into one PFGE cluster (cluster A), while other strains had less similarity (Fig. 1).

### MLST typing

**Fig. 2:**
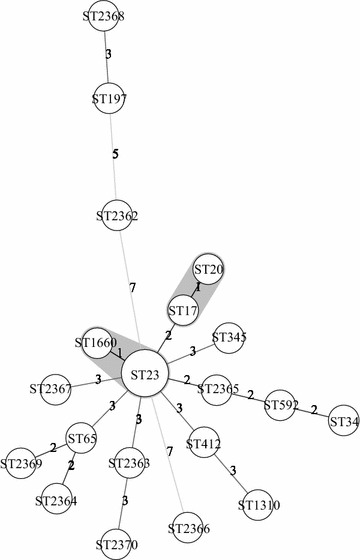
Minimum spanning tree of MLST typing for 22 *K. pneumoniae* strains

MLST was performed for all 22 isolates to investigate the genetic relationships. MLST analysis revealed 20 different sequence types (STs), including three ST23 strains and one ST65. In addition to identifying ST23 and ST65 which are prevail in Asia countries, ST2362, ST2363, ST2364, ST2365, ST2366, ST2367, ST2368, ST2369, and ST2370 were discerned for the first time. In the minimum spanning tree, there were two clone groups: one clone group contained ST23 and ST1660, and the other clone group contained ST17 and ST20 (Fig. 2).

## Discussion

*K. pneumoniae* is one of the most common Gram-negative pathogen found in human’s nasopharynx and in the intestinal tract [1]. It existed with 60–70% the carrier rate in the hospital environment, and was notified as a common pathogen caused nosocomial infection [1]. However, the community-acquired *K. pneumoniae* infection was rare reported. This study, through inclusion of *K. pneumoniae* into the existing diarrhea-syndrome surveillance, was able to detect *K. pneumoniae* infection in community and its risk for possible persistent transmission in population in Beijing.

The detection rate of *K. pneumoniae* in stool specimens from outpatients with diarrhea syndromes was about 0.5% (22/4340), which further demonstrated the existence of the community-acquired *K. pneumoniae* infection [11]. The prevalence in this study was much lower than the previous detection rate of *K. pneumoniae* in stool samples ranges from 5 to 38% in hospital patients [11], which emphasized the importance of nosocomial infection control as well necessary vigilance for detecting community-acquired *K. pneumoniae*.

Less similarity of the strains typed by PFGE or MLST indicated the unlikely long-term transmission of *K. pneumoniae* in the community. This study identified ST23 as the most common genotype, which were reported in many previous hospital-acquired infection studies in Asia [12, 13]. Noticeable this study isolated one ST65 strain, a mucoid phenotype and harbored *rmpA* gene for aerobactin, which more likely caused community-acquired infection [14, 15] and indicated as one of the independent risk factors for bacteremia in patients with pneumonia [13]. Moreover, the microbiological character of newly detected ST2362, ST2363, ST2364, ST2365, ST2366, ST2367, ST2368, ST2369, and ST2370, these strains presented clinical character are not thoroughly explicit, which needs further studies.

No ESBL production strains existed from stool specimens of diarrhea-syndrome outpatients in recent years illustrated that the community-acquired *K. pneumoniae* was not a serious public health problem. All the strains were sensitive to some antibiotics (e.g., cephalosporins, quinolones, and fluoroquinolones) universally in use for clinical treatment. However, hospital-acquired *K. pneumoniae* infection with resistance to multiple antibiotics agents has been increasing [16]. The *Klebsiella* species strains (e.g., TEM-type and SHV-type ESBLs, CTX-M type ESBLs) caused several predominant nosocomial infections [17]. This study did not identify the drug resistance for *K. pneumoniae* strains, but the diarrhea-syndrome surveillance identified serious drug resistance for *Shigella* spp. [4]. Given the consideration of horizontal transfer of drug resistance related genes as one of the most important mechanisms for the dramatically quickly dissemination of multi drug resistance among bacteria [18], monitoring the drug resistance of the community-acquired *K. pneumoniae* strains can provide a significant signal of drug resistance in population.

## Conclusions

The detection rate of *K. pneumoniae* in stool specimens from outpatients with diarrhea syndromes was about 0.5% in Beijing. Less similarity of the isolated strains indicated the unlikely long-term transmission of *K. pneumoniae* in the community. ST23 was the most common genotype. Drug resistance of the community-acquired *K. pneumoniae* was not a serious problem in comparing with hospital-acquired infections. High vigilance in the drug resistance of the community-acquired *K. pneumoniae* strains can provide a significant signal of drug resistance in population.

## Abbreviations

CLSI: Clinical and Laboratory Standards Institute
ESBL: extended-spectrum beta-lactamases
*K. pneumoniae*: *Klebsiella pneumoniae*
MIC: minimum inhibitory concentration
MLST: multilocus sequence typing
PFGE: pulsed field gel electrophoresis
STs: sequence types
UPGMA: the unweighted pair-group method
